# Long noncoding RNA profiling unveils LINC00960 as unfavorable prognostic biomarker promoting triple negative breast cancer progression

**DOI:** 10.1038/s41420-024-02091-3

**Published:** 2024-07-23

**Authors:** Ramesh Elango, Vishnubalaji Radhakrishnan, Sameera Rashid, Reem Al-Sarraf, Mohammed Akhtar, Khalid Ouararhni, Nehad M. Alajez

**Affiliations:** 1 Translational Oncology Research Center (TORC), Qatar Biomedical Research Institute (QBRI), Hamad Bin Khalifa University (HBKU), Qatar Foundation (QF), PO Box 34110, Doha, Qatar; 2 Department of Laboratory Medicine and Pathology (DLMP), Hamad Medical Corporation (HMC), Doha, Qatar; 3 The Christie NHS Foundation Trust, Manchester, United Kingdom; 4 Genomics Core Facility, Hamad Bin Khalifa University, Qatar Foundation, Doha, P.O. Box 34110, Qatar; 5 College of Health & Life Sciences, Hamad Bin Khalifa University (HBKU), Qatar Foundation (QF), Doha, Qatar

**Keywords:** Breast cancer, Long non-coding RNAs

## Abstract

Long noncoding RNAs (lncRNAs) play a critical role in breast cancer pathogenesis, including Triple-Negative Breast Cancer (TNBC) subtype. Identifying the lncRNA expression patterns across different breast cancer subtypes could provide valuable insights into their potential utilization as disease biomarkers and therapeutic targets. In this study, we profiled lncRNA expression in 96 breast cancer cases, revealing significant differences compared to normal breast tissue. Variations across breast cancer subtypes, including Hormone Receptor-positive (HR + ), HER2-positive (HER2 + ), HER2 + HR + , and TNBC, as well as in relation to tumor grade and patients’ age at diagnosis were observed. TNBC and HER2+ subtypes showed distinct clustering, while HER2 + HR+ tumors clustered closer to HR+ tumors based on their lncRNA profiles. Our data identified numerous enriched lncRNAs in TNBC, notably the elevated expression of LINC00960, which was subsequently validated in two additional datasets. Analysis of LINC00960 expression in an independent TNBC cohort (n = 360) revealed elevated expression of LINC00960 to correlate with cell movement, invasion, proliferation, and migration functional categories. Depletion of LINC00960 significantly reduced TNBC cell viability, colony formation, migration, and three-dimensional growth, while increasing cell death. Mechanistically, transcriptomic profiling of LINC00960-depleted cells confirmed its tumor-promoting role, likely through sponging of hsa-miR-34a-5p, hsa-miR-16-5p, and hsa-miR-183-5p, leading to the upregulation of cancer-promoting genes including BMI1, KRAS, and AKT3. Our findings highlight the distinct lncRNA expression patterns in breast cancer subtypes and underscore the crucial role for LINC00960 in promoting TNBC pathogenesis, suggesting its potential utilization as a prognostic marker and therapeutic target.

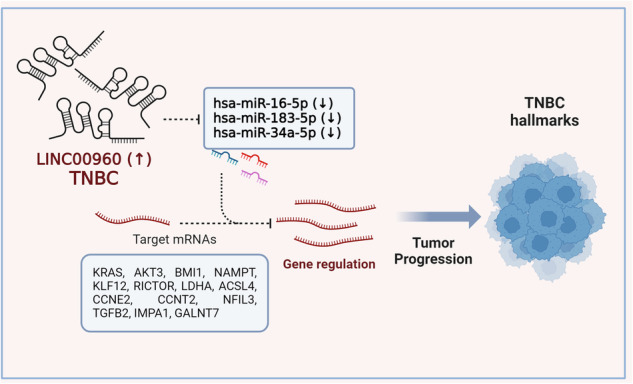

## Introduction

Triple-negative breast cancer (TNBC) presents one of the most aggressive forms of breast cancer, known for its high relapse rates and poor prognosis [1]. Despite advancements in cancer research, TNBC remains a significant challenge due to its complex molecular landscape and limited choices of targeted therapies. This urgency underscores the necessity for identifying novel biomarkers and therapeutic targets for TNBC.

Over the past decades, long non-coding RNAs (lncRNAs) have emerged as pivotal regulators of gene expression and cellular processes. LncRNAs, which are characterized by length exceeding 200 nucleotides, are involved in transcriptional, post-transcriptional, and epigenetic regulation [2]. Human GENCODE estimates suggest the existence of 16,000 to 100,000 lncRNA genes, with a substantial number of lncRNAs being implicated in various diseases, including cancer [3, 4]. These molecules exhibit tissue-specific expression patterns and play critical roles in oncogenesis, influencing cell proliferation, migration, invasion, apoptosis, and metastasis [5, 6].

Our previous research has contributed to the understanding of lncRNA expression patterns and functions in breast cancer [5, 7]. Building on this foundation, our current study aims to explore the lncRNA expression landscape in breast cancer patients, focusing on the identification of novel prognostic factors and therapeutic targets for TNBC.

Our data revealed the lncRNA transcriptional portrait in breast cancer. Through the integration of multiple datasets, we identified LINC00960 as an unfavorable prognostic marker for TNBC. Suppressing LINC00960 expression in TNBC cells significantly reduced cell proliferation and migration, increased cell death, and inhibited organotypic growth under three-dimensional (3D) culture conditions. These findings suggest that LINC00960 plays a crucial role in promoting oncogenic functions in TNBC and provides a potential target for therapeutic intervention, paving the way for improved prognosis and treatment strategies in TNBC.

## Results

### LncRNA profiling in breast cancer molecular subtypes compared to normal breast tissue

**Fig. 1:**
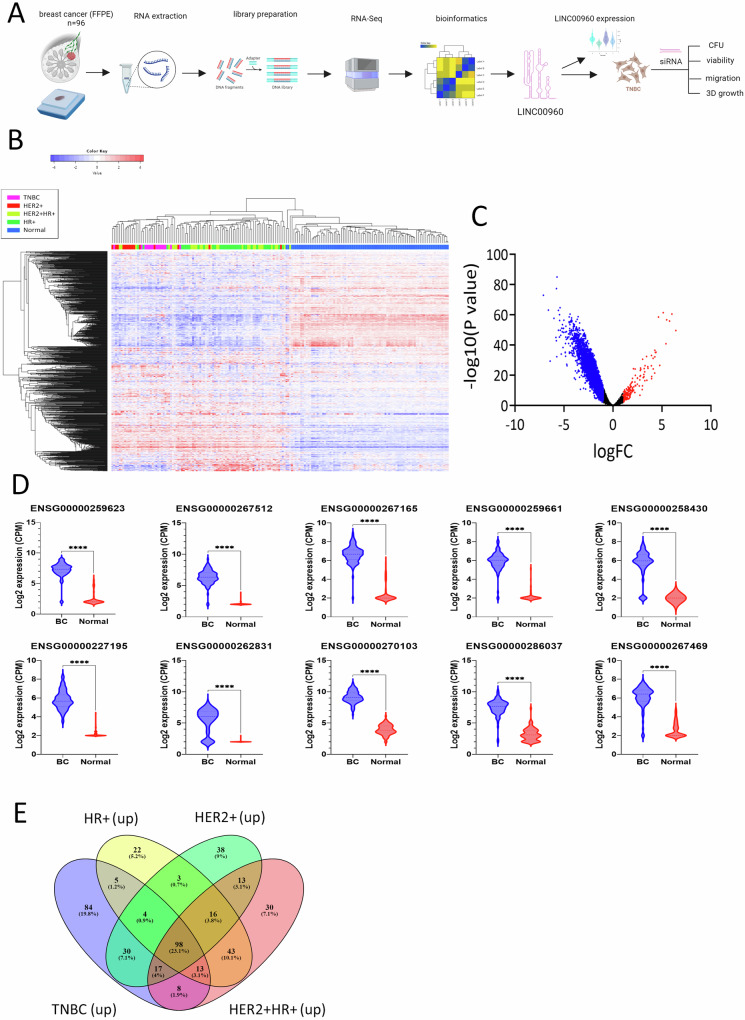
Hierarchical clustering of breast cancer based on lncRNA expression. **A** Schematic presentation of experimental workflow of the study. **B** Heatmap depicting clustering of 96 breast cancer patients and 88 normal as a function of breast cancer molecular subtype (TNBC, HR + , HER2 + , HR + HER2 + ) based on top 1000 most variable lncRNAs. Hierarchical clustering was conducted using correlation distance and average linkage. The color scale depicts the expression level of each gene. Each row represents a lncRNA gene, and each column represents a sample. **C** Volcano plot illustrating the upregulated (red) and downregulated (blue) lncRNAs in breast cancer vs. normal. **D** Violin plot depicting the expression of top 10 upregulated lncRNAs in breast cancer (*n* = 96) compared to normal (*n* = 88). **** *p* < 0.0001. **E** The overlap in upregulated lncRNAs in each breast cancer molecular subtype is depicted as a venn diagram.

In this study, we conducted a comprehensive analysis of lncRNA expression profiles in 96 well-characterized breast cancer samples, categorized into distinct molecular subtypes, including Hormone Receptor Positive (HR+), Human Epidermal Growth Factor Receptor 2 Positive (HER2 + ), HER2 + HR+ dual-positive, and TNBC. Figure 1A illustrates the comprehensive experimental methodology employed for lncRNA profiling and functional validation in our study cohort. To establish a baseline for comparison, we utilized RNA-Seq data from 88 normal breast tissue samples obtained from the PRJNA486023 dataset. Hierarchical clustering based on the expression of the top 1000 variable lncRNAs unveiled clear distinctions in the clustering patterns. Notably, all subtypes formed distinct clusters separate from the normal breast tissue, while TNBC and HER2+ clustered the farthest (Fig. 1B). The differentially expressed lncRNAs in breast cancer compared to normal tissue are depicted as volcano plot in Fig. 1C. Remarkably, our analysis revealed a predominance of downregulated lncRNAs in BC compared to normal tissue, consistent with our recent publication [8]. Figure 1D depicts the expression of the top 10 upregulated lncRNAs shown as a violin plot. For a more detailed insight into the differentially expressed lncRNAs, we provide comprehensive lists in Table S1-S4, highlighting the alterations specific to each molecular subtype when compared to normal tissue. Focusing on the upregulated lncRNAs across all breast cancer molecular subtypes, we identified a common set of 98 lncRNAs, as depicted in Fig. 1E and Table S5. Furthermore, our analysis unveiled subtype-specific signatures, with 84, 22, 38, and 30 lncRNAs selectively upregulated in TNBC, HR + , HER2 + , and HER2 + HR+ subtypes, respectively (Fig. 1E and Table S5).

These findings shed light on the complex lncRNA expression patterns in various breast cancer molecular subtypes, providing valuable insights into the molecular basis of breast cancer and potential avenues for further investigation.

### Differential analysis of LncRNA expression across breast cancer molecular subtypes and tumor grades

**Fig. 2:**
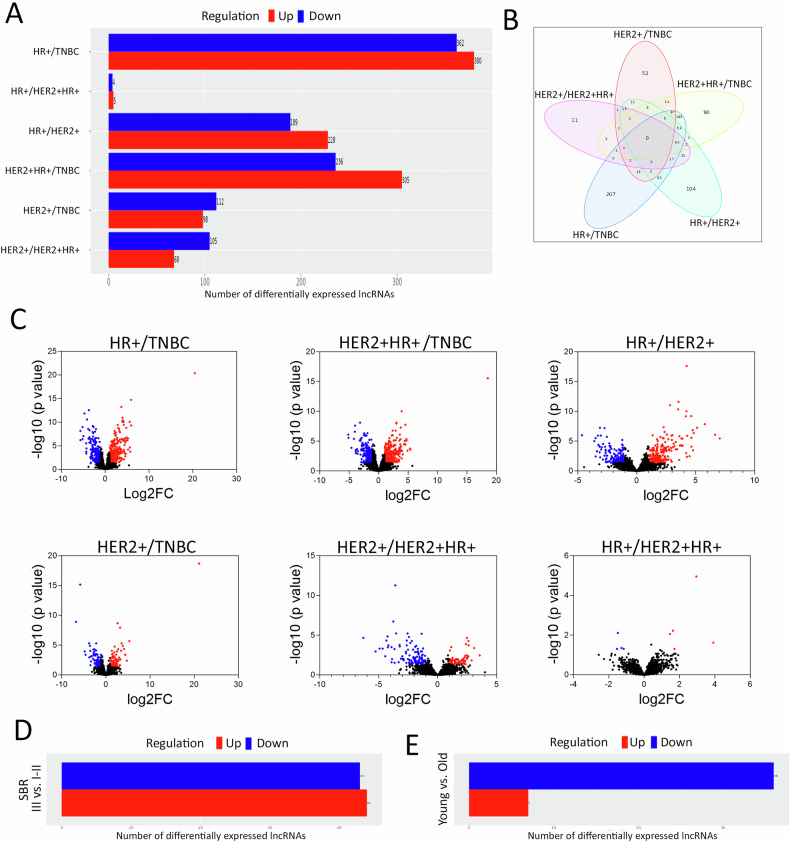
Differential lncRNA expression analysis as a function of breast cancer molecular subtypes, tumor grade, and age. **A** Bar chart depicting the number of differentially expressed lncRNAs in HR+ vs. TNBC, HR+ vs. HER2 + HR + , HR+ vs. HER2 + , HER2 + HR+ vs. TNBC, HER2+ vs. TNBC, and HER2+ vs. HER2 + HR+ after adjusting for age (young (≤40) vs. Old (>40)) and tumour grade (SBR III vs. SBR I-II) using 2.0-fold change (FC) and adjusted FDR (*p* < 0.05). **B** Venn diagram depicting the overlap between differentially expressed lncRNA in the indicated comparisons. **C** volcano plots depicting the upregulated (red) and downregulated (blue) lncRNAs in each comparison. **D** Bar chart depicting the number of differentially expressed lncRNAs in tumor SBR III vs. SBR I-II after adjusting for molecular subtype and age using 2.0-fold change (FC) and adjusted FDR (p < 0.05). **E** Bar chart depicting the number of differentially expressed lncRNAs in young (≤40) vs. Old (>40) patients after adjusting for molecular subtype and grade using 2.0-fold change (FC) and adjusted FDR (*p* < 0.05).

In our investigation, we carried out a comprehensive analysis of lncRNA expression profiles with a focus on discerning differences among distinct breast cancer molecular subtypes, while accounting for pertinent covariates such as tumor grade and age. Our results, as illustrated in Figs. 2A and B, highlighting the most prominent variations in lncRNA expression when comparing HR+ tumors versus TNBC, followed by the comparisons of HR + HER2+ versus TNBC, HR+ versus HER2 + , HER2+ versus TNBC, HER2+ versus HR + HER2 + , and HR+ versus HR + HER2 + . To provide a comprehensive visual representation of these differences, volcano plots portraying the differentially expressed lncRNAs in each comparison are presented in Fig. 2C.

Subsequently, our inquiry aimed at identifying lncRNA transcripts exhibiting enrichment in advanced tumor grades (SBR III) in contrast to lower grades (SBR I-II), which hold potential as prognostic biomarkers. After accounting for molecular subtypes and age as covariates, our analysis unveiled 44 upregulated and 43 downregulated lncRNAs in breast cancer SBR III versus SBR I-II, as depicted in Fig. 2D. A comprehensive list of these differentially expressed lncRNAs can be found in Table S6. Furthermore, we extended our investigation to assess the influence of patients’ age on lncRNA expression profiles. After adjusting for molecular subtype and tumor grade, our analysis identified 7 upregulated and 36 downregulated lncRNAs in younger (<40 years) compared to older (≥40 years) breast cancer patients, as elucidated in Fig. 2E. A comprehensive list of differentially expressed lncRNAs in breast cancer, stratified by patients’ age, can be found in Table S7. These findings underscore the multifaceted interplay between lncRNA expression, molecular subtypes, tumor grades, and patients’ age.

### Differential expression analysis unveils selective enrichment of LINC00960 in TNBC

**Fig. 3:**
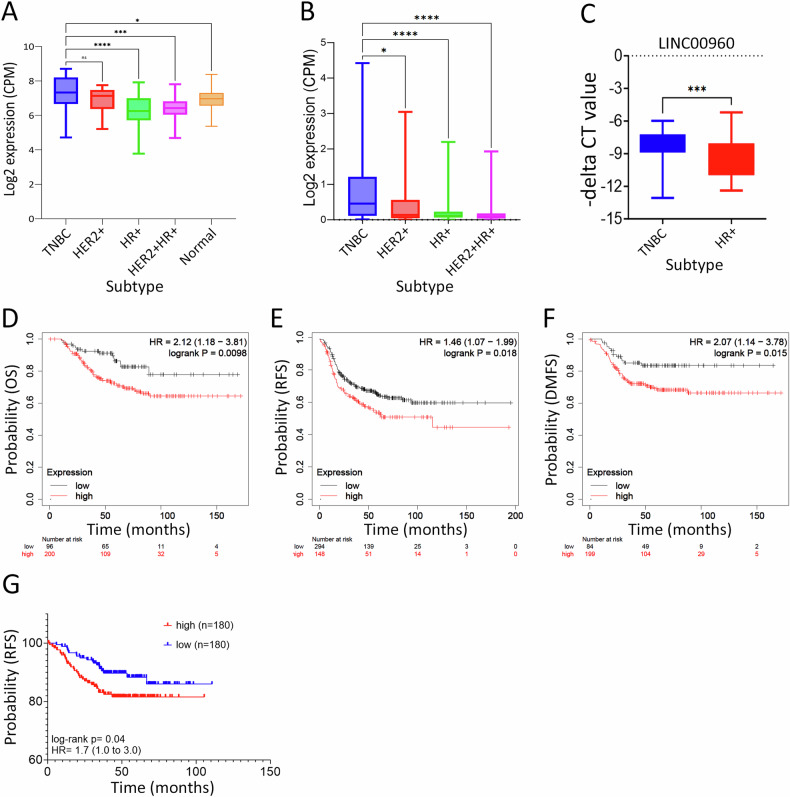
LINC00960 is enriched TNBC. **A** Expression of LINC00960 in TNBC *(n* = 15), HER2+ (*n* = 15), HR+ (*n* = 35), and HER2 + HR+ (*n* = 31) compared to normal (*n* = 88). Data are presented as box and whisker. *p < 0.1, ***p < 0.0001, ****p < 0.00001. **B** Validation of LINC00960 expression in TNBC (*n* = 88), HER2^+^ (*n* = 33), HR^+^ (*n* = 351), and HER2^+^HR^+^ (*n* = 99) from the TCGA BRCA dataset. **C** Validation of LINC00960 expression in TNBC (*n* = 9) compared to HR+ (*n* = 9) using RT-qPCR. Presentation of Kaplan–Meier overall survival **(**OS**, D)**, relapse-free survival **(**RFS**, E)** and distant metastasis-free survival **(**DMFS**, F)** analysis based on LINC00960 expression in breast cancer patients stratified into high vs low according to median expression. Kaplan–Meier RFS in a large cohort (*n* = 360) of TNBC based on median LINC00960 expression.

In the pursuit of understanding the lncRNA expression patterns within breast cancer subtypes, our analysis revealed enrichment of LINC00960 in the TNBC molecular subtype and upregulated expression compared to normal breast tissue, as presented in Fig. 3A. To further affirm this observation, we extended our investigation to a broader context by examining the expression patterns of LINC00960 across diverse breast cancer subtypes within the TCGA BRCA dataset, encompassing TNBC (*n* = 88), HER2+ (*n* = 33), HR+ (*n* = 351), and HR + HER2+ (*n* = 99) cases. Our findings, illustrated in Fig. 3B, consistently revealed elevated expression of LINC00960 in TNBC, further affirming its association with this aggressive subtype. Seeking additional validation, we explored the expression of LINC00960 in the TANRIC database. In accordance with our current findings, heightened expression of LINC00960 was observed within the basal subtype (Figure S1). The elevated expression of LINC00960 in TNBC compared to HR+ breast cancer was further validated in our cohort using RT-qPCR (Fig. 3C). Notably, our investigation extended further into the clinical implications of LINC00960. Survival analysis uncovered compelling evidence, with higher expression of LINC00960 strongly associated with adverse clinical outcomes in TNBC. Specifically, elevated expression in basal breast cancer correlated with poorer overall survival (OS) [HR = 2.12 (1.18–3.81), *p* = 0.0098], diminished distant metastasis-free survival (DMFS) [HR = 2.07 (1.14–3.78), *p* = 0.015], and reduced relapse-free survival (RFS) [HR = 1.46 (1.07–1.99), *p* = 0.018], as depicted in Figs. 3D, E, and F, respectively. Concordantly, elevated expression of LINC00960 also correlated with worse RFS in a large cohort of TNBC (*n* = 360), Fig. 3G. These findings collectively underscore the pivotal role for LINC00960 in TNBC and its significant implications in breast cancer prognosis, further underscoring its potential as a valuable prognostic biomarker and therapeutic target in the clinical management of this aggressive subtype.

### Expression of LINC00960 correlates with an oncogenic state in TNBC

**Fig. 4:**
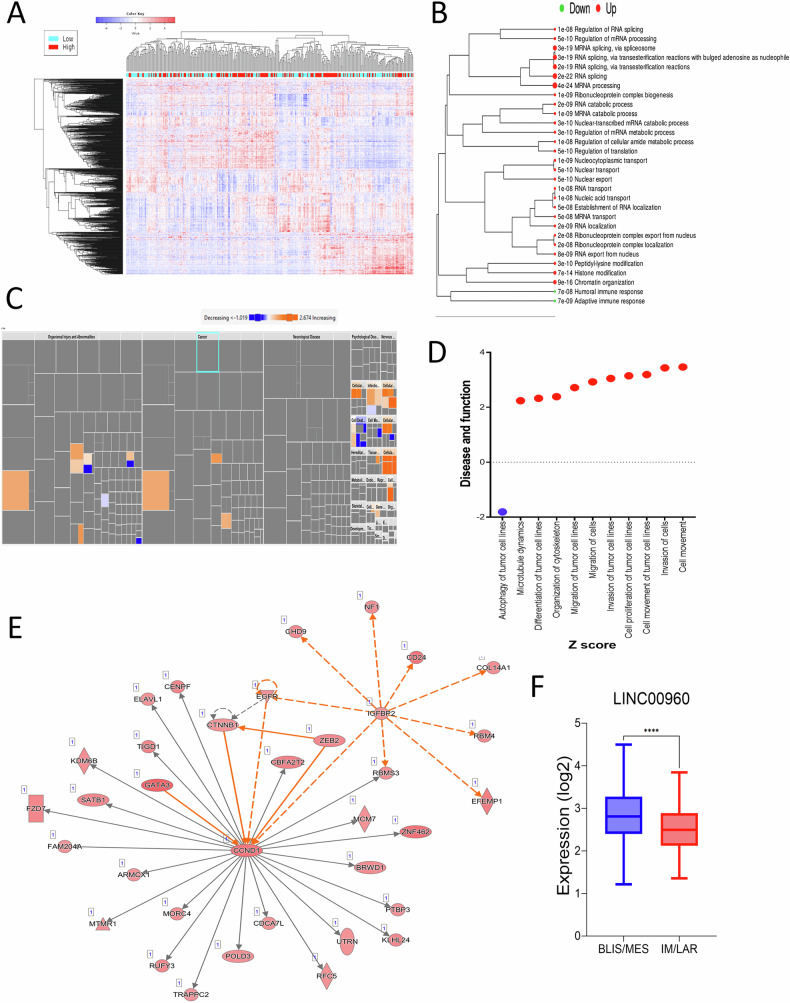
LINC00960 expression correlates oncogenic state in TNBC. **A** Hierarchical clustering of 360 TNBC based on LINC00960 median expression (high vs. low). **B** Pathway enrichment plot based on differentially expressed genes in LINC00960^high^ vs. LINC00960^low^ TNBC. **C** Disease and function map depicting activated (orange) and suppressed (blue) functional categories in LINC00960^high^ vs. LINC00960^low^ TNBC using IPA analysis. **D** Top enriched (red) and suppressed (blue) functional categories in LINC00960^high^ vs. LINC00960^low^. **E** Top enriched network based on differentially expressed genes in LINC00960^high^ vs. LINC00960^low^ TNBC. **F** Expression of LINC00960 in MES and BLIS compared to IM and LAR in TNBC subtypes. BLIS: basal-like immunosuppressed, MES: mesenchymal, IM: immunomodulatory, LAR: luminal androgen receptor.

In our quest to unravel the potential functional role for LINC00960 within the context of TNBC, we embarked on a comprehensive analysis involving a cohort of 360 TNBC patients. This cohort was stratified into two distinct groups based on the median expression levels of LINC00960, denoted as LINC00960^high^ and LINC00960^low^. Subsequently, we compared the corresponding protein-coding transcriptomes of these two groups, aiming to elucidate the underlying mechanisms associated with LINC00960 expression. Our analysis revealed distinctive clustering patterns based on LINC00960 expression, as illustrated in Fig. 4A. A comprehensive list of these differentially expressed genes based on median LINC00960 expression is provided in Table S8. To delve deeper into the functional consequences of LINC00960 expression, we conducted pathway enrichment analysis using differentially expressed genes between LINC00960^high^ and LINC00960^low^ TNBC subgroups (Fig. 4B). Intriguingly, the pathway enrichment plot prominently highlighted pathways associated with RNA processing and splicing in LINC00960^high^ TNBC, underscoring its potential regulatory role in this crucial biological process. Employing IPA, Fig. 4C provides a comprehensive disease and function map, delineating activated (in orange) and suppressed (in blue) functional categories in LINC00960^high^ versus LINC00960^low^ TNBC. Affected canonical pathways and matched entities provided in Table S9. Notably, our analysis unveiled a robust enrichment of functional categories related to cell movement, cell invasion, cell proliferation, cell migration, cytoskeleton organization, cell differentiation, and microtubule dynamics in LINC00960^high^ TNBC (Fig. 4D). In contrast, functional categories associated with cell death and autophagy were notably suppressed in this subgroup. To further substantiate our findings, we explored the top enriched network, as visually represented in Fig. 4E. Consistent with our findings, heightened expression of LINC00960 was observed in the aggressive MES and BLIS TNBC subtypes (Fig. 4F). Collectively, our comprehensive analysis sheds light on the pivotal role of this lncRNA where our findings revealed heightened expression of LINC00960 in the aggressive TNBC subtypes, correlating with more aggressive TNBC phenotype.

### Functional characterization of LINC00960 in TNBC

**Fig. 5:**
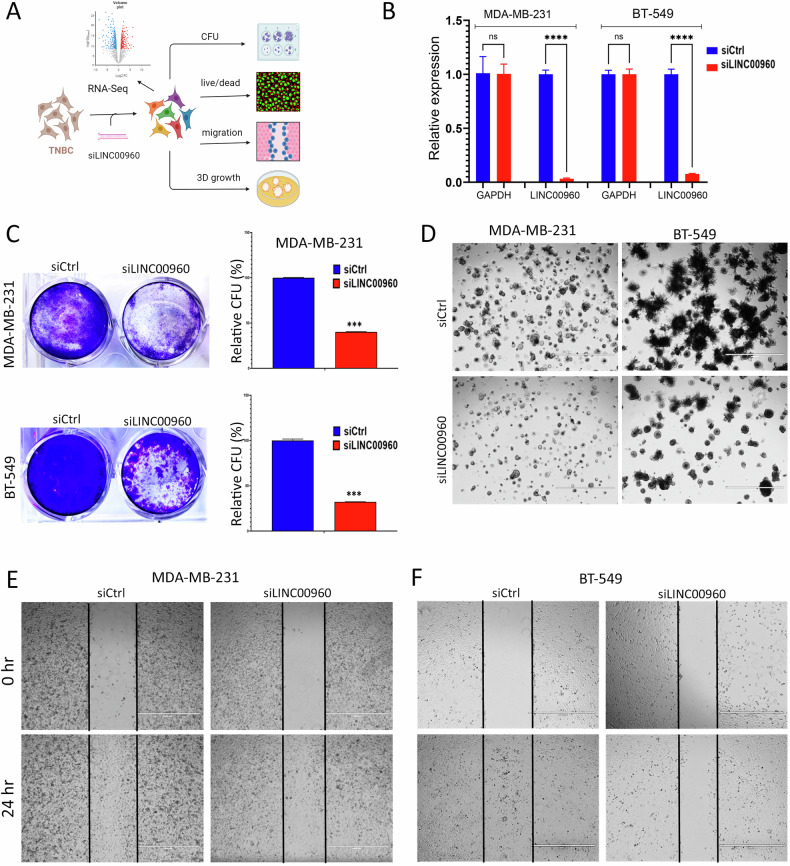
Depletion of LINC00960 attenuates TNBC cell proliferation and migration. **A** Schematic presentation of functional study workflow. **B** Signification inhibition of LINC00960 expression in TNBC models using siRNA-mediated gene silencing. Data are presented as mean ± S.D, *n* = 6. **C** CFU potential of MDA-MB-231 and BT-549 cells after siRNA-mediated LINC00960 suppression. The right panels show the quantification of CFU potential from same experiment. Data are presented as mean ± S.D, *n* = 4. **D** Inhibition of 3D colony organoid formation in MDA-MB-231 and BT-549 TNBC models in response to LINC00960 depletion. (Scale bar = 1000uM). Inhibition of cell migration in MDA-MB-231 **(E)** and BT-549 **(F)** after LINC00960 silencing.

**Fig. 6:**
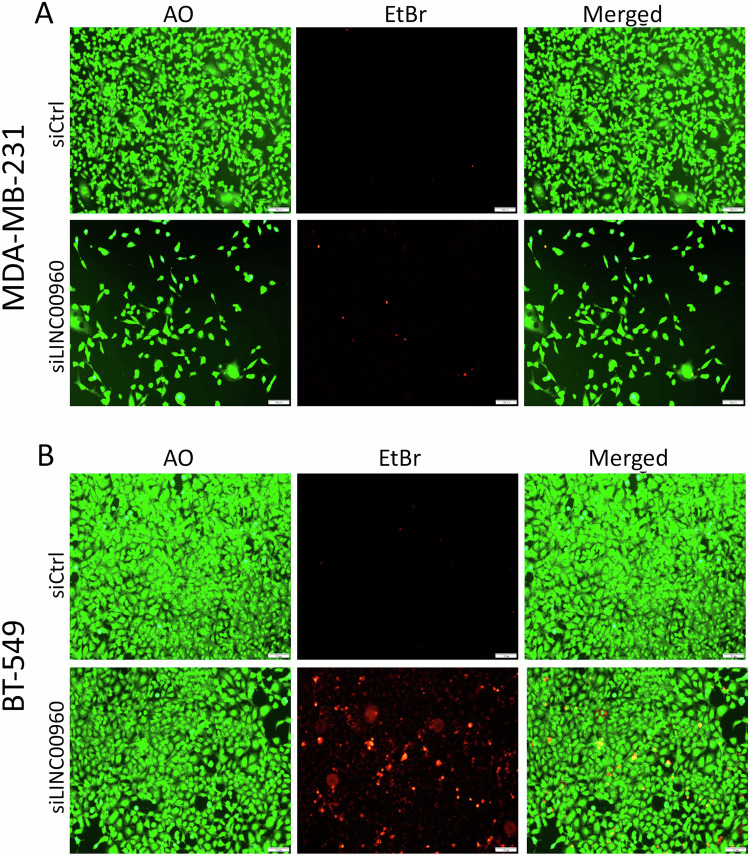
Dead-live staining of TNBC models in response to LINC00960 suppression. Representative live and dead staining of MDA-MB-231 **(A)** and BT-549 **(B)** in response to LINC00960 depletion. (Scale bar = 100uM). AO: acridine orange, EtBr: Ethidium bromide.

To elucidate the functional role of LINC00960 in TNBC, we conducted a series of loss-of-function studies employing siRNA targeting LINC00960 transcript in TNBC models. Figure 5A illustrates the experimental design to investigate the role of LINC00960 in TNBC. Our RT-qPCR data revealed substantial suppression of LINC00960 expression in TNBC models transfected with siLINC00960 compared to control cells (Fig. 5B). The results, as depicted in Fig. 5C, provided compelling evidence for the pivotal role for LINC00960 in promoting the CFU potential of both MDA-MB-231 and BT-549 TNBC models. Quantifications of CFU formation under different experimental conditions are shown on the right panels. Consistently, our investigations extended to the evaluation of organotypic growth of TNBC cells under 3D conditions (Fig. 5D). These findings unveiled a notable reduction in the number of colonies formed by siLINC00960-treated cells, along with a less densely packed colony formation compared to siControl treated cells, underscoring the involvement of LINC00960 in this critical aspect of TNBC biology. The wound healing assay highlighted suppression of cell migration in MDA-MB-231 (Fig. 5E) and BT-549 (Fig. 5F) TNBC cells post suppression of LINC00960 expression. Employing live/dead staining, our data further highlighted the profound impact of LINC00960 suppression on cell proliferation and increased cell death in both MDA-MB-231 (Fig. 6A) and BT-549 (Fig. 6B) TNBC cells. These functional observations are consistent with our earlier findings employing IPA, further implicating LINC00960 in the regulation of TNBC proliferation and migration, thereby enhancing our understanding of its functional significance in the context of TNBC.

### Molecular alterations in LINC00960-depleted TNBC

**Fig. 7:**
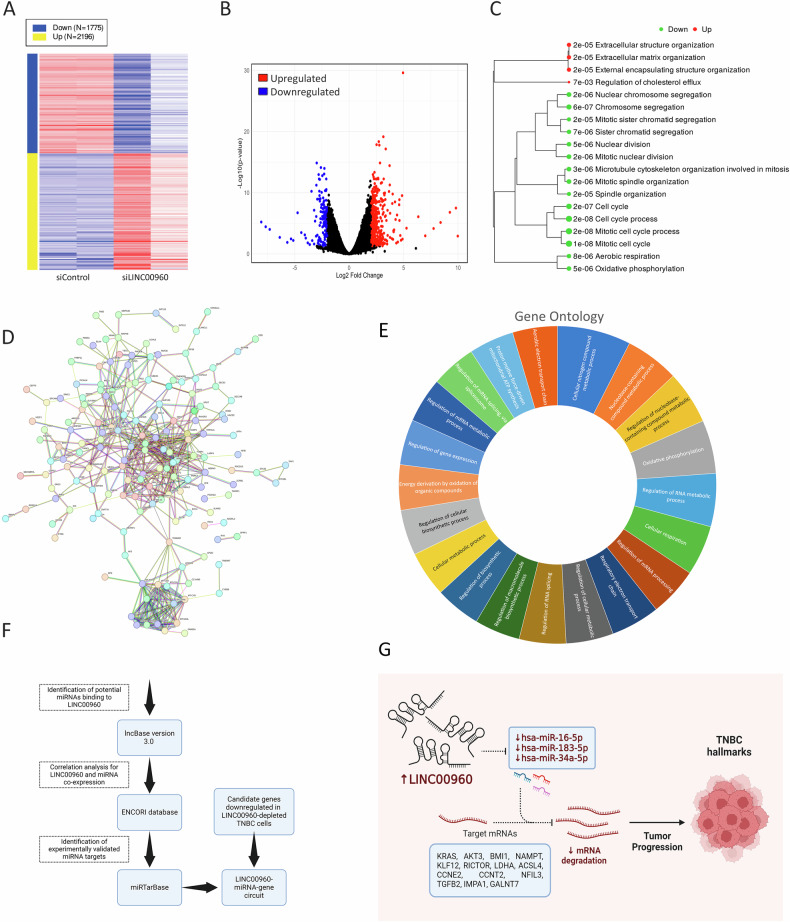
Multiple affected cancer hallmarks in LINC00960-depleted TNBC model. **A** Heatmap depicting differentially expressed genes in MDA-MB-231 cells transfected with siLINC00960 compared to siControl treated cells (2.0 fc, FDR < 0.05). **B** Volcano plot depicting upregulated (red) and downregulated (blue) genes in siLINC00960 compared to siControl cells. **C** Enrichment tree among differentially expressed genes. **D** STRING PPI network analysis on the 224 common genes. **E** Sunburst plot illustrated the top 20 enriched functional categories among the 224 common genes. **F** Schema depicting the strategy used to identify potential LINC00960-miRNA-gene circuits. **G** Schema depicting oncogenic role for LINC00960 in TNBC through sponging of hsa-miR-16-5p, hsa-miR-183-5p, and hsa-miR-34a-5p and subsequent upregulation of the indicated targets.

To gain a deeper insight into the mechanistic role of LINC00960 in TNBC, we conducted a whole transcriptome analysis on LINC00960-depleted TNBC cells. The heatmap depicted in Fig. 7A illustrates the differentially expressed genes in LINC00960-depleted TNBC cells (2.0 fc, FDR *p* < 0.05). A volcano plot depicting the upregulated (in red) and downregulated (in blue) genes in siLINC00960 compared to control siRNA-treated cells is shown (Fig. 7B). Our findings from gene ontology-based enrichment analysis, as presented in Fig. 7C, indicate a significant suppression of cellular processes related to cell cycle and oxidative phosphorylation among differentially expressed genes. Integration of transcripts upregulated in LINC00960^high^ TNBC and those suppressed in LINC00960-depleted TNBC models, we identified 224 genes associated with a dense network through STRING PPI network analysis, thereby validating our functional data (Fig. 7D). Top 20 enriched gene ontology functional categories in LINC00960-depleted TNBC cells are illustrated as sunburst plot in Fig. 7E. To better understand the mechanism by which LINC00960 could potentially impact TNBC biology, we integrated the DIANA database for miRNA-lncRNA interactions and identified eight microRNAs (hsa-miR-103a-3p, hsa-miR-1307-5p, hsa-miR-15a-5p, hsa-miR-16-5p, hsa-miR-183-5p, hsa-miR-23a-3p, hsa-miR-23b-3p, and hsa-miR-34a-5p) as potential targets for LINC00960 as depicted in the schema (Fig. 7F). We subsequently validated the correlation between LINC00960 and the eight miRNAs expression using the ENCORI database and identified hsa-miR-16-5p, hsa-miR-183-5p, and hsa-miR-34a-5p as potential targets for LINC00960 in breast cancer. Thirdly, we shortlisted the experimentally validated (reporter assay, western blot, or RT-qPCR) gene targets for those three miRNAs using the miRTarBase and identified 74 targets for hsa-miR-16-5p, 28 targets for hsa-miR-183-5p, and 128 targets for hsa-miR-34a-5p. When crossing the those validated targets with genes downregulated in LINC00960-depleted cells, we identified ACSL4, IMPA1, LDHA, CCNE2, NAMPT, KLF12, GALNT7, TGFB2, and CLOCK for hsa-miR-34a-5p; CCNT2, KRAS, AKT3, CD274, WT1, and SKAP2 for hsa-miR-16-5p; NFIL3 and PTEN for hsa-miR-183-5p, while BMI1 was identified as gene target for hsa-miR-16-5p and hsa-miR-183-5p. RICTOR was identified as gene target for hsa-miR-16-5p and hsa-miR-34a-5p. Taken together, this analysis identified LINC0960-miRNA-mRNA axis promoting TNBC cancer hallmarks (Fig. 7G).

These comprehensive analyses contribute to a more thorough understanding of LINC00960-mediated biological functions. Taken together, our analysis provides compelling evidence implicating LINC00960 as unfavorable prognostic biomarkers affecting several key Cancer hallmarks.

## Conclusion

Our study provides a comprehensive analysis of lncRNA expression in breast cancer, uncovering distinct patterns in each subtype. Notably, the aggressive TNBC subtype exhibited heightened expression of LINC00960 based on multiple breast cancer cohorts, associated with critical cellular processes. Targeted depletion of LINC00960 inhibited TNBC viability, migration, and growth while promoting cell death, confirming its potential oncogenic role. These findings suggest LINC00960 as a potential prognostic marker and therapeutic target for TNBC, offering avenues for novel treatment strategies.

## Discussion

Our study conducted comprehensive lncRNA profiling of breast cancer patients from the MENA region and identified LINC00960 as TNBC-associated lncRNA. The findings were extended to additional cohorts affirming LINC000960 as unfavorable prognostic biomarker promoting TNBC pathogenesis. In the Arab region, breast cancer incidence rates are on the rise, marked by distinct clinicopathological features, including variations in tumor size, molecular differences, hormone receptor subtypes, and age at first diagnosis. Arab women, with a median age of 48, often exhibit ER-negative tumors with poorly differentiated histological features and overexpression of HER2 [9]. Disparities within Arab populations extend beyond clinicopathological characteristics, evidenced by genomic studies revealing differences at the molecular level [10, 11]. Despite the wealth of genetic data from the USA, Europe, and other populations, research in Arab breast patients remains limited.

To address this gap of knowledge, our current study employs high-throughput lncRNA transcriptome analysis in female breast populations from this region, thus providing comprehensive lncRNA expression atlas in primary breast cancer from the MENA populations, where aggressive forms are prevalent. Our findings highlight the involvement of LINC00960 in breast cancer progression by regulating multiple oncogenic networks, providing crucial insights into the molecular landscape of breast cancer in this region and beyond.

Moreover, our study reveals that LINC00960, previously associated with various cancers, exhibits elevated expression in TNBC, predicting poor prognosis, which underscores its potential as a novel therapeutic target. Our research unveils the positive link between LINC00960 and numerous cancer hallmarks. Loss of function studies affirmed these findings, demonstrating suppressed TNBC viability, colony formation, organotypic growth, cell migration, and induction of cell death. Our findings align with prior research implicating LINC00960 in the progression of osteosarcoma [12], pancreatic [13], and lung adenocarcinoma [14]. Huang et al., explored the role of exosomal lncRNAs in bladder cancer progression, focusing on LINC00960 and LINC02470. Exosomes from high-grade bladder cancer cells, which contain these lncRNAs, enhanced the aggressiveness and epithelial-mesenchymal transition in low-grade bladder cancer cells. Specifically, the exosomes increased cell viability, migration, invasion, and colony formation, activating key signaling pathways such as β-catenin, Notch, and Smad2/3. Knockdown of LINC00960 or LINC02470 in high-grade cells reduced these aggressive traits and signaling activations in low-grade recipient cells, thus suggesting that LINC00960 and LINC02470 could be valuable biomarkers for monitoring bladder cancer progression [15].

While the association between LINC00960 and tumorigenicity has been documented in various cancers, our study marks the pioneering effort to unveil its expression in relation to molecular subtypes of breast cancer and elucidate its functional role in TNBC. Building upon current data, our investigation revealed an elevated expression of LINC00960 specifically in the basal breast cancer subtype using PAM50 classifications in the TANRIC database. The elevated expression of LINC00960 was linked with several cellular processes in TNBC, including cell movement, invasion, proliferation, migration, cytoskeleton organization, differentiation, and microtubule dynamics. Further bioinformatic investigation suggested LINC00960 to promote tumor progression through sponging hsa-miR-16-5p, hsa-miR-183-5p, and hsa-miR-34a-5p. This sponging leads to the subsequent upregulation of BMI1, KRAS, AKT3, KLF12, and NAMPT, along with several other cancer-promoting genes, thus promoting a more aggressive phenotype.

While our study provides significant insights, it is important to acknowledge potential limitations. One primary limitation is the sample size, which, although comprehensive for a regional study, may not capture the full heterogeneity of TNBC across different populations. Additionally, the lack of matched normal tissue for comparison represents another limitation. While findings from this study were validated in TCGA, TANRIC, and other datasets, these findings need further validation in larger, independent cohorts to confirm the prognostic value of LINC00960 and its utility as a therapeutic target. Future research should focus on expanding the sample size and including diverse populations to enhance the generalizability of our results. Furthermore, exploring the therapeutic applications of targeting LINC00960 in preclinical settings will be crucial. Investigations into the mechanisms of LINC00960 interaction with other molecular pathways and its plausible role in resistance to current therapies could provide deeper insights into its therapeutic potential. Additionally, exploring other mechanisms of action, such as its interactions with RNA-binding proteins, warrants further research [16].

RNA therapeutics have recently shown great potential in advancing precision health [17]. By leveraging the complex functions of cellular RNA, this growing field offers numerous strategies to modulate gene expression and key molecular pathways to combat cancer. Among these strategies, antisense oligonucleotides (ASOs) are particularly effective for selectively targeting and influencing crucial molecular mediators involved in cancer progression. Although challenges like improving delivery methods and reducing off-target effects persist, the rapid progress in RNA therapeutics for cancer is promising for developing more precise, individualized, and effective treatments. Spinraza and Eteplirsen represent two examples of FDA-approved ASOs for the treatment of human disease, demonstrating the potential of RNA-based treatments in precision oncology.

Our study contributes valuable insights, affirming the oncogenic properties of LINC00960 in TNBC, further supported by existing literature across diverse cancer types. Nonetheless, our study provides comprehensive lncRNA catalogue of breast cancer from the MENA region, proposing LINC00960 as a promising prognostic marker and therapeutic target, particularly for TNBCs. Exploration of the prognostic value and therapeutic potential of additional lncRNAs identified from current study warrants further investigation.

## Methods

### Patient characteristics

Formalin-fixed paraffin-embedded (FFPE) tissue samples were collected from 96 female breast cancer patients from Hamad Medical Corporation, Doha, Qatar. Detailed clinical and pathological information regarding study cohort can be found in our previous publication [18].

### Ethics approval and consent to participate

Ethical approval was granted by HMC (MRC-01-19-142) and Qatar Biomedical Research Institute (QBRI-IRB 2020-09-035) for this study. Written informed consent was not required since the study utilized archived FFPE specimens. Research was conducted in accordance with the Declaration of Helsinki.

### Total RNA extraction from FFPE tissues

Total RNA was extracted from FFPE core punches using the RecoverAll™ Total Nucleic Acid Isolation Kit (Ambion Inc., Life Technologies, USA). The procedures were followed according to the manufacturer’s protocol with minor modifications as we previously reported [18]. The RNA samples were then stored at -80 °C.

### Quality assessment of RNA

The quality and quantity of the extracted RNA were assessed using on-chip electrophoresis. This was accomplished using the Agilent RNA 6000 Nano Kit (Agilent Technologies, CA, USA) and the Agilent 2100 Bioanalyzer (Agilent Technologies). Procedures were conducted in accordance with the manufacturer’s instructions.

### Total RNA library preparation and RNA sequencing

Total RNA was used to prepare libraries with the TruSeq Stranded Total RNA Library Kit (Illumina). First, 500 ng of total RNA underwent rRNA depletion. cDNA synthesis was then carried out, and the double-stranded cDNA was end-repaired and adenylated. Barcoded DNA adapters were added, and the cDNA was amplified. The library quality was assessed using the Agilent Bioanalyzer, and quantification was done with the Qubit system. Libraries that passed quality control were pooled and sequenced on the NEXTSEQ2000 system, achieving at least 50 million paired-end reads per sample. The RNA transcriptomic data are available in the SRA repository under BioProject number PRJNA954402.

### Total RNA‑Seq data analysis and bioinformatics

Paired-end FASTQ files were aligned to the GRCh38 reference genome using the built-in module in CLC Genomics Workbench v21.0.5 with default settings. Expression data (total count) were then imported into iDEP.951 and initially normalized to CPM (count per million, which adjusts for differences in sequencing depth). Data transformation was conducted using EdgeR (log2(CPM+c), a method for stabilizing variance), as previously described [19]. LncRNAs with a minimum expression of 1 CPM in at least 10 samples were retained. Hierarchical clustering was performed using correlation distance and average linkage. Differentially Expressed Genes (DEGs) were identified using DESeq2 iDEP.951.

### RNA-Seq on LINC00960-depleted TNBC cells

The MDA-MB-231 TNBC model was transfected with ON-TARGETplus SMARTpool siRNA targeting LINC00960 or scrambled negative siRNA control. Seventy-two hr later, cells were collected, and total RNA was extracted and was subjected to total RNA isolation and sequencing as described above. FASTQ data were then aligned to the GRCh38 reference genome using the built-in module in CLC Genomics Workbench v21.0.5 with default settings. Subsequent analyses were conducted using iDEP.951 as described above.

### Real-time quantitative polymerase chain reaction (RT-qPCR)

LINC00960 was subjected to RT-qPCR to validate its expression in samples from the same cohort. Briefly, 500 ng of RNA was reverse transcribed to cDNA using the high-capacity cDNA Reverse Transcription kit (Applied Biosystems, Foster City, CA, USA). RT-qPCR was done using LINC00960_F: GCAGCACCATATGAGAGGTT and LINC00960_R: TGGCATGCTGCAGAGATCAA; GAPDH_F: GGAGCGAGATCCCTCCAAAAT, and GAPDH_R: GGCTGTTGTCATACTTCTCATGG and the PowerUp™ SYBR™ Green Master Mix (Applied Biosystems) on QuantStudio 7/6 Flex qPCR (Applied Biosystems). Relative levels of transcripts were determined from their respective CT values normalized against GAPDH transcript levels and were presented as –(delta CT).

### Validation of LINC00960 expression and enriched functional categories using publicly available datasets

RNA-Seq data were retrieved from 360 TNBC and 88 normal tissue samples (accession no. PRJNA486023) from the Sequence Read Archive (SRA) database using the SRA toolkit v2.9.2, as previously described [20]. To estimate the expression of LINC00960 and associated protein-coding genes from this dataset, a Kallisto index was initially generated by creating a de Bruijn graph using the GENCODE release 33 reference assembly and using a k-mer length of 31. Subsequently, FASTQ files from the PRJNA486023 dataset were mapped and aligned to the generated Kallisto index. Differential expression and gene set enrichment analysis in LINC00960^high^ versus LINC00960^low^ TNBC were conducted using iDEP.951. Differentially expressed genes (1.5 FC and <0.1 FDR) were imported into Ingenuity Pathway Analysis (IPA) for canonical, upstream regulator, disease and function, and network analysis.

### Cell line authentication employing short tandem repeat (STR) analysis

Genomic DNA (gDNA) extracted from the MDA-MB-231 and BT-549 TNBC cell lines served as the input for STR profiling. Using the AmpFLSTR Identifiler PCR amplification kit (Thermo Fisher Scientific, Inc., Waltham, MA, USA), we amplified DNA, including positive and negative controls. Subsequently, PCR products were prepared for electrophoresis by introducing Hi-Di Formamide and a size standard mixture to each sample and allelic ladder. Electrophoresis was conducted using the Genetic Analyzer 3500xl DX system. Analysis of allelic calls was performed through the Gene Mapper software provided by Applied Biosystems/Thermo Fisher Scientific. Reference STR profiles were obtained from the American Type Culture Collection (ATCC) website (https://www.atcc.org/).

### Cell culture and transfection

Human TNBC cell lines MDA-MB-231 and BT-549 were grown in DMEM with 10% fetal bovine serum (FBS) and 1% penicillin/streptomycin (Pen-Strep) (Thermo Scientific, Rockford, IL, USA). The cells were maintained as monolayers at 37 °C with 5% CO2 in a humidified incubator. To study the role of LINC00960 in breast cancer, these cells were transfected with siLINC00960 or a scrambled control (Dharmacon, Cat. No # R-028057-00 and Cat. No # D-001320-10). Transfection was done using a reverse transfection method as described by [21]. Briefly, siLINC00960 (30 nM) was diluted in 50 µL of Opti-MEM (Gibco, Carlsbad, CA, USA), and 1.5 µL of Lipofectamine 2000 (Invitrogen) was also diluted in 50 µL of Opti-MEM. The mixtures were combined and incubated for 20 min at room temperature. Subsequently, 100 µL of this transfection mix was added to each well of a 24-well plate, followed by 300 µL of cell suspension (0.168 ×10^6^ cells/mL). After 20 hr, the transfection mixture was replaced with complete DMEM.

### Colony forming unit (CFU) assay

The CFU ability of MDA-MB-231 and BT-549 TNBC cells transfected with siLINC00960 or a scrambled siRNA negative control was determined using a clonogenic assay as previously described [18]. On day 7, the plates were washed twice in PBS and then stained with crystal violet (1%) for 10 min at room temperature. Images were captured and compared with those of the control. Subsequently, plates were air-dried at room temperature, and CFUs were quantified by dissolving crystal violet in 10% SDS and measuring absorbance at 590 nm. The experiments were repeated twice, and data are represented as mean ± SD from four technical replicates.

### Viability staining

Apoptosis and necrosis in siLINC00960 or scrambled siRNA control transfected TNBC cells were assessed using the acridine orange /ethidium bromide (AO/EtBr) fluorescence staining). On day 5 post siLINC00960 transfection, MDA-MB-231 and BT-549 cells were washed twice with PBS and subsequently stained with a dual fluorescent staining solution containing 100 mg/mL AO and 100 mg/mL EtBr (AO/EtBr, Sigma Aldrich, St. Louis, MO, USA) for 2 min. The cells were then observed and imaged under an Olympus IX73 fluorescence microscope (Olympus, Tokyo, Japan). Differential uptake of AO/EtBr allowed the identification of viable and non-viable cells, with AO used for visualizing apoptotic cells and EtBr for identifying necrotic cells.

### Wound healing (scratch) assay

To assess the migration of LINC00960-depleted cells, a scratch assay was performed as previously described [22]. Cells were trypsinized and re-seeded in 12-well plates on day 5 post-transfection. Once they reached confluence, cell monolayers were scratched using a sterile 200-μl pipette tip. After eliminating cell debris by washing adherent cells twice, the medium was replaced with fresh culture medium. Images of the wounded region were captured immediately (0 hr) and after 24 hr, using phase-contrast microscopy.

### Organoid dome culture

To generate 3D organoids, MDA-MB-231 and BT-549 cells were transfected with siLINC00960 or a scrambled siRNA negative control. Cell pellets (250,000 cells/mL) were mixed with overnight-thawed Matrigel (Corning; 356231; Growth Factor Reduced (GFR) Basement Membrane Matrix) as previously described [22]. Subsequently, multiple drops of cell suspension were plated in pre-warmed (37 °C) 60-mm ultra-low attachment culture dishes (Corning; 3261). The plates were then placed upside-down in a 37 °C, 5% CO2 cell culture incubator to allow the droplets to solidify for 20 min before adding 4-5 mL of expansion medium. Organoid formation was observed under the microscope after one week.

### Identification of LINC00960 microRNA (miRNA) and gene network

To identify potential miRNA and gene targets for the LINC00960 circuit, we firstly utilized the lncBase version 3.0 database (https://diana.e-ce.uth.gr/lncbasev3) to compile a list of miRNAs that could potentially bind to LINC00960. Next, we investigated the miRTarBase database (https://mirtarbase.cuhk.edu.cn/~miRTarBase/miRTarBase_2022/php/index.php) to determine the experimentally validated gene targets of the identified microRNAs. Specifically, we focused on hsa-miR-16-5p, hsa-miR-183-5p, and hsa-miR-34a-5p, and retrieved their gene targets validated through reporter assays, Western blot, or RT-qPCR. Subsequently, we cross-referenced these validated gene targets with a list of genes that were downregulated in LINC00960-depleted TNBC cells. This comparison allowed us to identify the final LINC00960-miRNA-gene circuit, through integration of data from both lncBase and miRTarBase with our gene expression analysis.

### Statistical analysis

Statistical analyses for differentially expressed lncRNAs were conducted in iDEP.951. Fold change (2.0) and FDR adjusted p value <0.05 was used as cutoff, unless stated otherwise. Survival analyses were conducted in IBM SPSS v26.0 and log-rank p value of <0.05 was considered significant. Graphing and pairwise statistical analyses were conducted in GraphPad prism v9.

### Supplementary information

Supplementary tables

Figure S1

## Data Availability

Processed data are provided in supplementary tables. The RNA transcriptomic data are available in the SRA repository under BioProject number PRJNA954402.
