# Supplementary material for: Long noncoding RNA profiling unveils LINC00960 as unfavorable prognostic biomarker promoting triple negative breast cancer progression
Source: Cell Death Discov. 2024 Jul 23;10:333. doi: 10.1038/s41420-024-02091-3 (PMC11263344; doi:10.1038/s41420-024-02091-3)
Supplement: Supplementary file 2 — Figure S1 [file 41420_2024_2091_MOESM2_ESM.pdf]

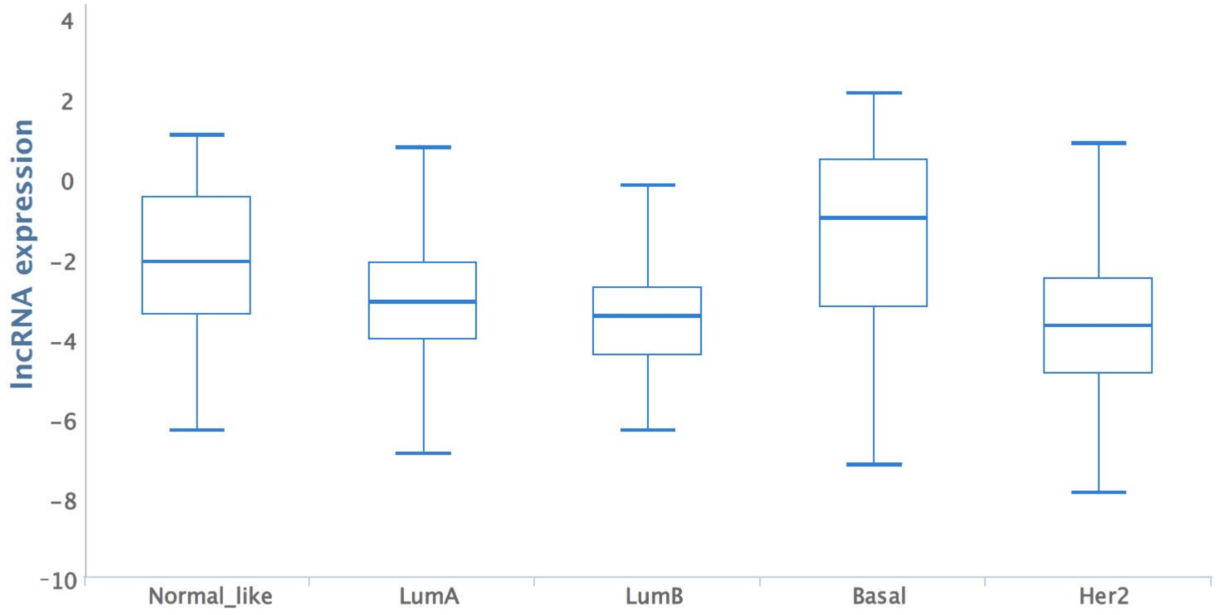

Figure S1. Expression of LINC00960 in different breast cancer subtypes. Data was retrieved from TANRIC database (https://bioinformatics.mdanderson.org/public-software/tanric/).
